# Perinatal and Neonatal Outcomes of Refugee Infants in a Tertiary Hospital in Turkey

**DOI:** 10.7759/cureus.44917

**Published:** 2023-09-08

**Authors:** Gonca Vardar, Eren Ozek

**Affiliations:** 1 Department of Pediatrics, Division of Neonatology, Marmara University School of Medicine, Istanbul, TUR

**Keywords:** perinatal, turkey, refugee, outcome, neonate

## Abstract

Background

Worldwide, the immigration problem has been increasing due to conflicts. In recent years, Turkey accepted more than 3.8 million refugees from many countries, principally Syria.

Aims

In this study, we aimed to evaluate the prenatal features and neonatal outcomes of refugees and Turkish controls hospitalized in a tertiary neonatal intensive care unit in Turkey.

Materials and methods

This retrospective case-control study included comparative data related to populations based on whether they were refugees or not. Their perinatal and neonatal "outcomes" were compared.

Results

Among the 254 analyzed neonates, 127 were born to refugee mothers, and 127 controls were born to non-refugee Turkish mothers. The refugee rate in our hospitalized neonates was nine, a young mother’s age (p=0.010) with a higher rate of adolescent pregnancies at OR 2.78 (95% CI 0.96-8.05) (p=0.032), and consanguineous marriage at OR 0.57 (95% CI 0.32-1.02) (p=0.031) in comparison to non-refugees. The incidence of ABO incompatibility-related hemolytic jaundice (p=0.013) was higher in the refugees. The rate of formula feeding in the first month of life was significantly higher at OR 0.49 (95% CI 0.25-0.92) (p=0.027) in neonates born to refugee mothers. Despite lower perinatal care rates in refugees at OR 7.23 (95%CI 4.12-12.69) (<0.001), preterm morbidities did not differ between refugees and non-refugee preterm infants ≤32 gestational age (p>0.05).

Conclusion

The importance of breast milk must be strongly encouraged to initiate and promote exclusive breastfeeding for the infants of refugees. Race is still an important risk factor for ABO incompatibility-related hemolytic jaundice. Providing high-quality healthcare is sufficient to prevent worse outcomes in refugee neonates.

## Introduction

Refugees are individuals who have had to flee their hometowns because of war, persecution, or violence. Due to its location as a major transit country between Asia and Europe, Turkey is the largest refugee-hosting country, with 3.8 million refugees that comprise 15% of individuals displaced across borders [1].

Refugee groups are reported to have a greater rate of adolescent pregnancy and preterm births [2,3]. Additionally, it has been noted that diverse ethnic refugee groups in resettlement nations have a higher risk for gestational diabetes mellitus despite having a lower cesarean delivery rate when compared to women living in receiving countries [4,5]. The literature has reported variable outcomes in refugee infants concerning gestational age, perinatal asphyxia, jaundice, low birth weight, respiratory distress syndrome, meconium aspiration, prolonged hospitalization, lower Apgar scores, and assisted ventilation [2,6,7].

The study aimed to assess the perinatal characteristics of refugee mothers and the incidence of neonatal morbidities in refugee newborns.

## Materials and methods

Study design and ethical considerations

This retrospective case-control study used the data of neonates admitted to the NICU of Marmara University Research and Training Hospital in Istanbul between January 2019 and July 2022. Perinatal and neonatal characteristics of refugee and Turkish neonates were compared. The Marmara University School of Medicine's Clinical Studies Ethics Committee approved the study (Date: 22.07. 2022 and No: 09.2022.997).

Participants

Our study participants were gathered from medical records based on whether they were refugees, and their perinatal and neonatal "outcomes" were compared. All infants delivered to refugee or native Turkish mothers who required hospitalization in our hospital's NICU during the study period were evaluated for study inclusion. The study’s control group was composed of Turkish neonates with a similar week of gestation who were admitted to the NICU in the same period to reduce the possibility of affecting neonatal survival from the gestational week. Almost at the same time, the first native case admitted to NICU with a similar week of gestation was included in the study, following the hospitalized refugee neonate. The study population comprised neonates admitted to the NICU who were born at our hospital or admitted to our outpatient clinics or emergency rooms. Patients with incomplete data were excluded from the study.

Data collection and definitions

Data were extracted from patients’ electronic medical records. The demographic features of refugee and Turkish mothers and neonates were noted and analyzed. We recorded nationality, gender, gestational age (GA), birth weight (BW), small-for-GA (SGA), large-for-GA (LGA), head circumference, and height at birth. The following data were noted: Apgar scores at the first and fifth minutes, delivery mode, consanguineous marriages, antenatal steroid use, maternal disease and smoking status, maternal age, gravidity, parity, perinatal care, the incidence of >18h preterm premature rupture of the membranes (PPROM), assisted reproductive therapy, maternal hepatitis B surface antigen positivity, congenital malformation, duration of stay, and mortality. Perinatal care included follow-up visits and ultrasonography evaluations. Primary prenatal follow-up of pregnant women in Turkey is conducted in the first 14 weeks, between 18 and 24, 30 and 32, and 36 and 38 weeks.

The following were accepted as neonatal morbidities: respiratory distress syndrome (RDS), transient tachypnea of the newborn (TTN), sepsis, jaundice, polycythemia, perinatal asphyxia, meconium aspiration syndrome (MAS), pneumonia, congenital heart disease, metabolic disease, hypoglycemia, congenital tumors, coronavirus disease (COVID-19), and Hirschsprung’s disease.

Small for gestational age (SGA) was defined as infants having BW under the 10th percentile. LGA infants were also defined as having a BW over the 90th percentile for their GA, respectively. The growth of babies with height and head circumference at birth was calculated according to the INTERGROWTH-21st standards [8]. GA was calculated based on the Ballard scoring system or the first day of the last menstrual period. The evaluation of neonates admitted with jaundice was based on serum bilirubin concentration using the American Academy of Pediatrics recommendations [9]. Sepsis was accepted as being early-onset sepsis (EOS) if it appeared within the first 72 hours of life and late-onset sepsis (LOS) if it presented beyond three days of age [10]. The serum glucose concentration threshold in symptomatic hypoglycemic infants in the first 24 hours of life was accepted as 40mg/dL. In asymptomatic newborns, this value was accepted as being 25 mg/dL for up to four hours, 35 mg/dl for between 4 and 24 hours, 50 mg/dL from between 24 and 48 hours, and 60 mg/dL following 48 hours of life [11]. Venous hematocrit of >70% or ≥65%, accompanied by hypoglycemia, decreased muscle tone, respiratory and feeding difficulties, jitteriness, and seizures, were accepted as symptomatic neonatal polycythemia [12]. Perinatal asphyxia was defined as an Apgar score >5 at 10 min, or need for resuscitation >10 min, a cord blood pH ≤7.0, and/or a base deficit of ≤-16 [13].

Preterm morbidities (≤32 GA) were noted: hemodynamically significant patent ductus arteriosus (hsPDA), intraventricular hemorrhage (IVH), necrotizing enterocolitis (NEC), bronchopulmonary dysplasia (BPD), and retinopathy of prematurity (ROP). Hemodynamically significant patent ductus arteriosus (hsPDA) was evaluated using echocardiography, organ blood flow Doppler, clinical signs, and the need for treatment [14]. Periventricular/intraventricular hemorrhage (IVH) was classified according to Volpe’s cranial ultrasound classification [15]. Bronchopulmonary dysplasia (BPD) was defined according to oxygen requirement and ventilator support at 36 weeks of gestation [16]. Retinopathy of prematurity (ROP) was classified by standardized international criteria [17]. Bell’s criteria were used to define severe NEC [18].

Outcome measures

The primary outcome measures were neonatal morbidities and mortality.

Secondary outcome measurements included perinatal features (maternal age, gravidity, parity, perinatal care, delivery mode, multiple births, consanguineous marriages, antenatal steroid use, maternal disease, and smoking status, the incidence of >18h PPROM, assisted reproductive treatment, maternal hepatitis B surface antigen positivity), small-for-GA (SGA), large-for-GA (LGA), head circumference at birth, length at birth, Apgar scores for the first and fifth minutes, congenital malformation, need for resuscitation at birth, need for phototherapy, etiology of neonatal jaundice, surfactant administration, breastfeeding vs. formula feeding, duration of stay, preterm morbidities (RDS, PDA, IVH, ROP, BPD, and NEC), opposition to vaccination, and treatment costs.

Statistical analysis

Statistical Product and Service Solutions (SPSS) for Windows (IBM Corp. Released 2017, Version 25.0. Armonk, NY, USA) was used for statistical analysis. The sample size of the study was calculated with the “G*Power” 3.1.9.4 version. Overall, 206 (each group of 103) neonates are required for the study to estimate the results with 0.90 power (alpha=0.05, effect size=0.41). The Pearson chi-square test was used to compare categorical variables that were reported as n(%). Continuous variables were expressed using the mean ± standard deviation (SD) or medians and ranges (min-max). Using the Kolmogorov-Smirnov test, the normality of data for continuous variables was evaluated and then compared with either an unpaired Student's t-test or a Mann-Whitney U test. Statistical significance was considered when p<0.05 for all tests.

## Results

**Figure 1:**
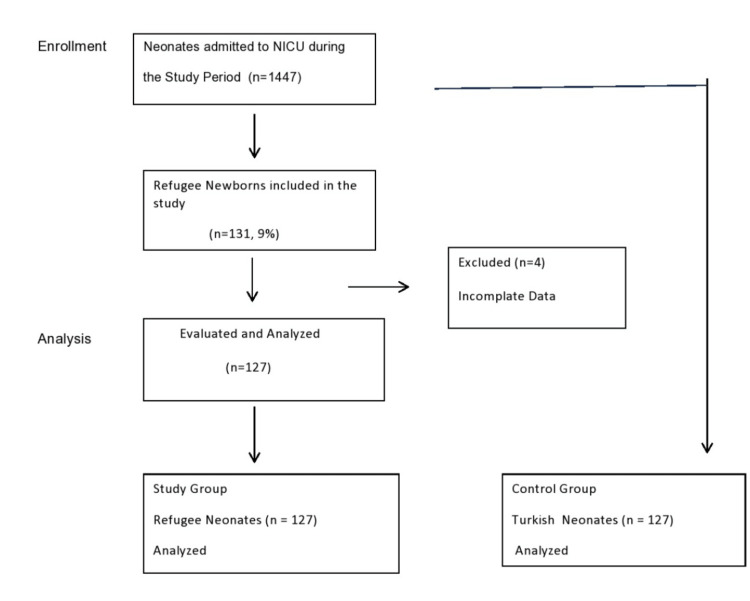
Flow chart for the selection of eligible infants in the study

During the study period, 1,447 infants were admitted to the NICU, 131 (9%) of them were born to refugee mothers. Four of these newborns were excluded because of incomplete data. Therefore, 127 neonates born to refugee mothers and 127 controls born to non-refugee Turkish mothers who met the study’s inclusion criteria were compared in Figure 1. The nationality of refugee mothers was Syrian (n=106, 83.5%), Asian (n=15, 11.8%), African (n=3, 2.4%), and others (n=3, 2.4%).

Table 1 shows the demographics of refugees and non-refugee Turkish mothers are shown. When refugee and non-refugee Turkish mothers were compared, maternal age (p=0.010), perinatal care (p<0.001), a cesarean delivery (p<0.001), and antenatal steroids (p=0.032) were significantly lower in refugee mothers’ babies. However, gravidity (p=0.002), parity (p=0.003), adolescent pregnancy (p=0.032), and consanguineous marriage (p=0.031) were significantly higher in the refugee group. There was no significant difference between the rates of assisted reproductive therapy (p=0.409), vaccine hesitancy and refusal (p=1.0), >18 hours PPROM (p=0.053), preeclampsia (p=0.062), gestational diabetes (p=0.425), and smoking (p=0.237) in the two groups.

**Table 1:** Demographic characteristics of refugees and non-refugee Turkish mothers IQR: interquartile range; HBs Ag: hepatitis B surface antigen; PPROM: preterm premature rupture of membranes; OR (95% CI): odds ratio (95% confidence interval)

		Study group (n=127)	Control group (n=127)	P	OR (95% C.I.)
Age (years), median (IQR)		26 (21-30)	28 (25-32)	0.010	
Adolescent pregnancy (16-19 years), n (%)		14 (11)	5 (3.9)	0.032	2.78 (0.96-8.05)
Gravidity, n (%)	1	29 (22.8)	45 (35.4)	0.002	1.85 (1.06-3.21)
	2	28 (22.0)	36 (28.3)		1.39 (0.79-2.47)
	3	23 (18.1)	25 (19.7)		1.10 (0.59-2.07)
	≥4	47 (37.0)	21 (16.5)		0.33 (0.18-0.60)
Parity, n (%)	1	35 (27.6)	52 (40.9)	0.003	1.82 (1.07-3.08)
	2	29 (22.8)	41 (32.3)		1.61 (0.92-2.81)
	3	28 (22.0)	17 (13.4)		0.54 (0.28-1.05)
	≥4	35 (27.6)	17 (13.4)		0.40 (0.21-0.77)
Assisted reproductive therapy, n (%)		2 (1.5)	4 (3.1)	0.409	2.03 (0.36-11.3)
Perinatal care, n (%)		27 (21.2)	84 (66.1)	<0.001	7.23 (4.12-12.69)
Cesarean delivery, n (%)		52 (40.9)	86 (67.7)	<0.001	3.02 (1.81-5.05)
HBs ag positivity, n (%)		1 (0.7)	3 (2.3)	0.313	3.04 (0.31-29.7)
Opposition to vaccination, n (%)		1 (0.7)	1 (0.7)	1.0	1 (0.06-16.16)
Antenatal steroids, n (%)		5 (3.9)	14 (11)	0.032	3.02 (1.05-8.66)
Consanguineous marriage, n (%)		40 (31.5)	25 (19.7)	0.031	0.57 (0.32-1.02)
>18h PPROM, n (%)		8 (6.3)	2 (1.5)	0.053	0.23 (0.05-1.14)
Preeclampsia, n (%)		4 (3.1)	11 (8.6)	0.062	2.91 (0.90-9.41)
Gestational diabetes mellitus, n (%)		6 (4.7)	9 (7)	0.425	1.53 (0.53-4.45)
Smoking, n (%)		4 (3.1)	8 (6.3)	0.237	2.07 (0.61-7.05)

Demographics and clinical characteristics of refugee and non-refugee Turkish newborns are demonstrated in Table 2. The formula feeding in the first month of life was significantly high (p=0.027), and exclusive breastfeeding (EB) was considerably lower (p=0.04) in neonates born to refugee mothers. There was no statistically significant difference between SGA (p=0.897) or LGA infants (p=0.897), male gender (p=0.379), resuscitation at birth (p=0.226), congenital anomalies (p=0.198), need for phototherapy (p=0.314), exchange transfusion (p=0.561), and mortality (p=1.00), gestational weeks at birth (p=0.99), birth weight (p=0.793), birth height (p=0.805), head circumference at birth (p=0,131), first- (p=0.806) and fifth- (p=0.598) minute Apgar scores, age of newborns at hospitalization (p=0.869), time to discharge (p=0.861), and median treatment costs (p=0.235) between infants born to refugee mothers and non-refugee Turkish mothers.

**Table 2:** Demographic and clinical characteristics of refugee and Turkish newborns IQR: interquartile range; SGA: small for gestational age; LGA: large for gestational age; OR (95% CI): odds ratio (95% confidence interval)

	Study group (n=127)	Control group (n=127)	P	OR (95% CI)
Gestational age, weeks, median (IQR)	38 (35-39)	38 (35-39)	0.99	
Birth weight, g, median (IQR)	2980 (2380-3475)	3100 (2270-3400)	0.793	
SGA, n (%)	10 (7.8)	11 (8.6)	0.897	1.10 (0.45-2.71)
LGA, n (%)	32 (25.2)	29 (22.8)	0.897	0.87 (0.49-1.56)
Birth height, cm, median (IQR)	49 (46-51)	49 (46-50)	0.805	
Head circumference at birth, cm, median (IQR)	34(32-35)	34 (32-35)	0,131	
Gender, male, n (%)	64 (50.3)	57 (44.8)	0.379	0.80 (0.48-1.31)
One-minute Apgar score, median (IQR)	8 (7-9)	8 (6-9)	0.806	
Five-minute Apgar score, median (IQR)	9 (8-10)	9 (8-10)	0.598	
Resuscitation at birth, n (%)	36 (28.3)	45 (35.4)	0.226	1.38 (0.81-2.35)
Age at hospitalization, days, median (IQR)	1 (1-1)	1 (1-1)	0.869	
Congenital anomalies, n (%)	9 (7)	15 (11.8)	0.198	1.75 (0.73-4.17)
Phototherapy, n (%)	63 (49.6)	55 (43.3)	0.314	0.77 (0.47-1.27)
Exchange transfusion, n (%)	2 (1.5)	1 (0.79)	0.561	0.49 (0.04-5.54)
Formula feeding, n (%)	32 (25.2)	18 (14.1)	0.027	0.49 (0.25-0.92)
Exclusive breastfeeding, n (%)	95 (74.8%)	109 (85.8%)	0.04	0.49 (0.25-0.92)
Time to discharge, days, median (IQR)	8 (5-17)	8 (5-18)	0.861	
Mortality, n (%)	4 (3.1)	4 (3.1)	1.00	1 (0.24-4.08)
Treatment costs, TL, median (IQR)	11.037.79 (4.383.86-2.7926.90)	13.393.13 (6.810.46-30.100.08)	0.235	

ABO incompatibility (p=0.013) related to hemolytic jaundice was significantly higher in refugee neonates. The direct Coombs test was positive in all refugee neonates with ABO incompatibility 100% (n=6) and have blood type A 83.3% (n=5). On the other hand, the other neonatal morbidities including meconium aspiration syndrome (p=0.053) were similar between the two groups of neonates (Table 3).

**Table 3:** Diagnosis of refugee and Turkish infants in NICU Odds ratio (95% confidence interval)

Diagnosis, n(%)	Study group (n=127)	Control group (n=127)	p	OR (95% CI)
Respiratory distress syndrome	18 (14.1)	18 (14.1)	1.00	1 (0.49-2.02)
Transient tachypnea of the newborn	36 (28.3)	47 (37)	0.141	1.48(0.87-2.51)
Early-onset sepsis	8 (6.3)	10 (7.8)	0.625	1.27 (0.48-3.33)
Late-onset sepsis	9 (7)	7 (5.5)	0.605	0.76 (0.27-2.12)
Prematurity (only)	1 (0.4)	5 (2)	0.09	5.16 (0.59-44.84)
Small for gestational age	1 (0.7)	0 (0)	0.316	
Meconium aspiration syndrome	8 (6.3)	2 (1.5)	0.053	0.23 (0.05-1.14)
Bronchopneumonia	5 (3.9)	2 (1.5)	0.250	0.39 (0.07-2.05)
SARS-CoV-2	2 (1.5)	2 (1.5)	1.00	1 (0.13-7.21)
ABO incompatibility with jaundice	6 (4.7)	0 (0)	0.013	
Rh incompatibility with jaundice	1(0.79)	0 (0)	0.316	
Neonatal jaundice	16 (12.6)	15 (11.8)	0.848	0.92 (0.43-1.97)
Congenital heart disease	4 (3.1)	4 (3.1)	1.00	1 (0.24-4.08)
Polycythemia	1 (0.79)	0 (0)	0.316	
Hypoglycemia	2 (1.5)	1 (0.79)	0.561	0.49 (0.04-5.54)
Syndromic disorders	5 (3.9)	10 (7.8)	0.183	2.08 (0.69-6.28)
Metabolic disease	2 (1.5)	1 (0.79)	0.561	0.49 (0.04-5.54)
Perinatal asphyxia	5 (3.9)	3 (2.3)	0.472	0.59 (0.13-2.52)
Dehydration, weight loss	5 (3.9)	5 (3.9)	1.00	1 (0.28-3.54)
Hirschsprung disease	2 (1.5)	0 (0)	0.156	
Congenital adrenal hyperplasia	1 (0.79)	0 (0)	0.316	
Solid tumors	0 (0)	2 (1.5)	1.00	

When refugee and non-refugee Turkish preterm infants ≤32 GA were compared, the median GA (p=(0.987), birth weight (p=0.169), male gender (p=0.298), time of discharge (p=0.364), mortality rate (p=0.070), RDS (p=0.423), hsPDA (p=0.678), (p=0.846), NEC (p=0.172), ROP (p=0.692), and BPD (p=0.373) were similar between refugee and non-refugee Turkish preterm infants (Table 4). Three of the native neonates (15.7%) required surgery versus one (6.2%) refugee neonate because of NEC (p=0.377).

**Table 4:** Demographic and clinical characteristics of ≤32 gestational weeks refugee and Turkish preterm infants IQR: interquartile range; RDS: respiratory distress syndrome; hsPDA: hemodynamically significant patent ductus arteriosus; IVH: intraventricular hemorrhage; NEC: necrotizing enterocolitis; ROP: retinopathy of prematurity; BPD: bronchopulmonary dysplasia; odds ratio (95% confidence interval)

	Study group (n=18)	Control group (n=18)	p	OR (95% CI)
Gestational age, weeks, median (IQR)	29 (28-32)	29 (28-32)	0.987	
Birth weight, g, median (IQR)	1432 (1245-1715)	1155 (879-1525)	0.169	
Gender, male, n (%)	8 (44.4)	5 (27.8)	0.298	0.48 (0.12-1.92)
RDS	13 (72.2)	15 (83.3)	0.423	1.92 (0.38-9.64)
hsPDA, n (%)	4 (26.6)	6 (33.3)	0.678	1.37 (0.30-6.20)
IVH, n (%)	2 (13.3)	2 (11.1)	0.846	0.81 (0.1-6.58)
NEC, n (%)	4 (26.6)	9 (50)	0.172	2.7 (0.63-11.97)
ROP, n (%)	6 (40)	6 (33.3)	0.692	0.75 (0.18-3.11)
BPD, n (%)	6 (40)	10 (55.5)	0.373	1.87 (0.46-7.52)
Time of discharge, days, median (IQR)	43 (26-81)	58 (35-69)	0.364	
Mortality, n (%)	0 (0)	3 (16.7)	0.070	

A subgroup analysis was conducted, including Turkish neonates (n=127) and Syrian refugees (n=106, 83.5%), who constitute most of the immigrant population. Meconium aspiration syndrome and >18h PPROM was significantly higher (7 (6.6%) vs. 2 (1.5%), p=0.047) in the Syrian population (8 (7.5%) vs. 2 (1.5%), (p=0.025)), respectively.

## Discussion

Turkey's refugee population has expanded over the years as it has been a transit point for those seeking to travel from Asia to Europe. Our study group mainly consisted of Syrian refugees [19]. Many factors influence the adverse birth outcomes of refugee mothers and neonates, including immigration status, cultural background, beliefs, educational status, financial situation in the host nation, social networks, and communication. In our study, pregnant refugees used prenatal care less than Turkish mothers, even though they have access. According to Vurgec et al., the primary hurdle appears to be language, followed by cultural differences, insufficient knowledge, and social support [20].

Our results showed that refugee mothers were younger and had a greater number of gravidity and parity when compared to non-refugee Turkish mothers. This could be related to social and cultural factors and the belief that marriage could provide protection in war. Adolescent pregnancy and consanguineous marriages were also higher in refugee pregnancies in our study, as reported previously in the literature [3]. This may be because our hospital is located in a region where refugees with low socioeconomic status come to settle. Among the refugee population, early-age consanguineous marriage for girls can assure economic status and protection from various assaults in a newly migrated country.

The cesarean delivery in refugee mothers was found to be lower when compared to Turkish mothers (40.9% vs. 67.7%). Although studies from Jordan and Turkey show that refugees have higher cesarean rates, our study found higher in Turkish mothers [21,22]. This is consistent with the worldwide increased rate of cesarean sections over the last decades, which is much beyond the WHO recommendation of 10-15% [23]. Higher medico-legal anxiety and a history of a cesarean section could explain why Turkish mothers have a higher number of cesarean sections. Vural et al. reported that gestational diabetes, PPROM, and hypertensive disorders of pregnancy were more common in Turkish women when compared to refugees [2]. However, Kandasamy et al. found that gestational diabetes, hypertension in pregnancy, and smoking did not differ between refugee and non-refugee pregnant [24]. The incidence of preeclampsia, gestational diabetes, >18h PPROM, HBsAg positivity, smoking, and assisted reproductive therapy were similar between refugee and non-refugee Turkish mothers in our study. This result can be ascribed to Turkey's improved prenatal care for refugee women.

Our study showed significantly lower perinatal care in refugee infants than in non-refugees. This finding may explain the significantly higher incidence of meconium aspiration syndrome and PPROM in Syrian immigrants. However, hospitalization due to preterm birth did not differ between refugee and resident populations. Research on Afghan, Syrian, and Iraqi refugees showed a greater incidence of LBW neonates [6,25]. However, our results do not support this finding. We observed no difference between refugee and Turkish neonates regarding GA, birth weight, and anthropometric measurements. APGAR scores tend to be lower, and the need for resuscitation at birth was higher in refugee infants than in residents [21]. However, the APGAR scores and the need for resuscitation were not statistically different between the two groups in our study. These findings may be associated with improved socioeconomic status and high healthcare systems for refugees in Turkey. There was no significant difference in congenital anomalies, age of admission, hospitalization period, phototherapy, and exchange transfusion between groups. The formula feeding in the first month of life was significantly higher, and exclusive breastfeeding (EB) was significantly lower in neonates born to refugee mothers in our study. This may result from insufficient knowledge resulting from a language barrier, young age, and economic issues, including transportation and nourishment for refugee mothers. We believe refugee women should be encouraged to breastfeed more frequently to initiate and promote EB.

According to a retrospective analysis conducted in refugee camps managed by the United Nations High Commissioner for Refugees, infant mortality ranged from 12 to 50 fatalities per 1,000 births [26]. This was close to the 3.1% infant mortality among refugees in our nation. Likewise, 3.1% was the mortality of non-refugee Turkish controls.

Most morbidities at NICU admission did not differ between the two groups in our study. There are contradictory findings in the literature about the prevalence of prenatal hypoxia in refugee neonates [3,6]. Differences in regional conditions in access to health services in Turkey may be one reason for this. Kimyon et al. reported that the incidence of ROP did not differ between refugee and native populations [27]. Also, Silahli et al. found no significant difference in the incidence of NEC, IVH, PDA, and ROP between immigrants and residents, which was consistent with our findings [3]. In a study comparing the incidence of RDS and BPD between refugee and non-refugee Turkish neonates, RDS was found to be similar. The multifactorial etiology of these morbidities can explain this. However, a significantly higher BPD was reported in the Turkish population. The same study stated that the time to discharge was significantly higher in the refugee population [6]. In our study, a comparison of infants ≤ 32 GA showed similar results in preterm morbidities. The discharge time did not differ. The discordance in the prevalence of BPD and time to discharge in our study may be attributed to the small number of preterm infants with ≤32 GA.

Studies suggest that race is a risk factor for ABO hemolytic disease [28]. In a study, among blood group, A infants born to group O mothers, the prevalence of a positive direct Coombs test was reported to be highest in Asians [29]. Since race has been removed from the "New Hyperbilirubinemia Guideline 2022," there has been a debate regarding race-conscious treatment and potential harms [30]. ABO incompatibility with jaundice was higher in NICU-admitted refugee neonates compared to non-refugee Turkish controls in our study. Direct Coombs test was positive in all refugee neonates with ABO incompatibility. The majority of them were infants with blood type A. Our results showed that race remains an important risk factor for ABO incompatibility-related hemolytic jaundice.

Our research has several limitations. Due to its retrospective design and communication problems with immigrants, data were limited to available from records. The study was undertaken in a tertiary referral center where refugees can receive significantly improved healthcare compared to those in camps or other parts of Turkey. Despite these limitations, our study shows that providing high-quality healthcare is crucial to prevent worse outcomes in refugee neonates. If these refugee mothers receive even better perinatal care by overcoming the language problem, the EB can be increased. The increase in EB can reduce the death and illness of children in our century of wars and migrations.

## Conclusions

The differences in maternal and neonatal outcomes between refugees and residents help identify the problems the vulnerable immigrant population faces. The lack of difference in major morbidities was partly attributed to equal management protocols based on unit policies. Breastfeeding should be encouraged among refugee mothers to prevent formula feeding. Race is still an important risk factor for ABO incompatibility-related hemolytic jaundice. Even though the Turkish government has provided refugees with adequate healthcare and socioeconomic conditions, international support is needed to enhance financial and political conditions and education.
